# TiO_2_ Nanocomposite Coatings and Inactivation of Carbapenemase-Producing Klebsiella Pneumoniae Biofilm—Opportunities and Challenges

**DOI:** 10.3390/microorganisms12040684

**Published:** 2024-03-28

**Authors:** Alina-Simona Bereanu, Bogdan Ioan Vintilă, Rareș Bereanu, Ioana Roxana Codru, Adrian Hașegan, Ciprian Olteanu, Vicențiu Săceleanu, Mihai Sava

**Affiliations:** 1 Faculty of Medicine, Lucian Blaga University of Sibiu, Lucian Blaga Street 2A, 550169 Sibiu, Romania; alina.bereanu@ulbsibiu.ro (A.-S.B.); rares.bereanu@ulbsibiu.ro (R.B.); adrian.hasegan@ulbsibiu.ro (A.H.); vicentiu.saceleanu@ulbsibiu.ro (V.S.); mihai.sava@ulbsibiu.ro (M.S.); 2 County Clinical Emergency Hospital, Bld. Corneliu Coposu nr. 2-4, 550245 Sibiu, Romania; ciprian.laborator@yahoo.com

**Keywords:** carbapenemase-producing *Klebsiella pneumoniae*, KPC-producing *Klebsiella pneumoniae*, carbapenem-resistant *Klebsiella pneumoniae* biofilm, biofilms, TiO_2_ nanoparticles, TiO_2_ nanocomposite coatings

## Abstract

The worldwide increase of multidrug-resistant Gram-negative bacteria is a global threat. The emergence and global spread of *Klebsiella pneumoniae* carbapenemase- (KPC-) producing *Klebsiella pneumoniae* represent a particular concern. This pathogen has increased resistance and abilities to persist in human reservoirs, in hospital environments, on medical devices, and to generate biofilms. Mortality related to this microorganism is high among immunosuppressed oncological patients and those with multiple hospitalizations and an extended stay in intensive care. There is a severe threat posed by the ability of biofilms to grow and resist antibiotics. Various nanotechnology-based strategies have been studied and developed to prevent and combat serious health problems caused by biofilm infections. The aim of this review was to evaluate the implications of nanotechnology in eradicating biofilms with KPC-producing *Klebsiella pneumoniae*, one of the bacteria most frequently associated with nosocomial infections in intensive care units, including in our department, and to highlight studies presenting the potential applicability of TiO_2_ nanocomposite materials in hospital practice. We also described the frequency of the presence of bacterial biofilms on medical surfaces, devices, and equipment. TiO_2_ nanocomposite coatings are one of the best long-term options for antimicrobial efficacy due to their biocompatibility, stability, corrosion resistance, and low cost; they find their applicability in hospital practice due to their critical antimicrobial role for surfaces and orthopedic and dental implants. The International Agency for Research on Cancer has recently classified titanium dioxide nanoparticles (TiO_2_ NPs) as possibly carcinogenic. Currently, there is an interest in the ecological, non-toxic synthesis of TiO_2_ nanoparticles via biological methods. Biogenic, non-toxic nanoparticles have remarkable properties due to their biocompatibility, stability, and size. Few studies have mentioned the use of nanoparticle-coated surfaces as antibiofilm agents. A literature review was performed to identify publications related to KPC-producing *Klebsiella pneumoniae* biofilms and antimicrobial TiO_2_ photocatalytic nanocomposite coatings. There are few reviews on the antibacterial and antibiofilm applications of TiO_2_ photocatalytic nanocomposite coatings. TiO_2_ nanoparticles demonstrated marked antibiofilm activity, but being nano in size, these nanoparticles can penetrate cell membranes and may initiate cellular toxicity and genotoxicity. Biogenic TiO_2_ nanoparticles obtained via green, ecological technology have less applicability but are actively investigated.

## 1. Introduction

*Klebsiella pneumoniae* is a pathogenic, non-motile bacterium that has been associated with ventilator-associated pneumonia (VAP), postoperative infections, and sepsis, possibly even leading to septic shock and death. The overall spread of multidrug-resistant Gram-negative bacteria is a worldwide threat. The development and global spread of multidrug-resistant (MDR) *Klebsiella pneumoniae* are of particular concern. MDR *K. pneumoniae* strains usually lead to hard-to-treat or untreatable nosocomial infections. The primary multidrug-resistant mechanism is enzyme production. Three major classes of enzymes are involved: Ambler class A (*Klebsiella pneumoniae* carbapenemase) (KPC), B (Metallo-beta-lactamase) (MBLs), and D (oxacillinases) (OXA-48-like). All of these enzymes are mediated via plasmids, mobile genetic elements carrying antibiotic-resistant genes, facilitating the dissemination of carbapenem resistance worldwide [1,2].

*Klebsiella pneumoniae* carbapenemase-producing *Klebsiella pneumonia* (KPC-Kp) are mostly encountered in Greece, Italy, Israel, the United States, Colombia, and Argentina [1,3,4]. In 2016, the Annual Report of the European Antibiotic Surveillance Network published an average percentage of carbapenem resistance of 6.1%, with a prevalence distribution in Greece, Italy, and Romania [4]. A study by the European Centre for Disease Prevention and Control and the National Public Health Organization in Greece (2022) reported a quick spread of carbapenemase-producing, highly drug-resistant *Klebsiella pneumoniae* (sequence type 39) [5].

This pathogen has increased resistance and the ability to persist in human reservoirs and in hospital environments, and to generate biofilms. Nosocomial spread of KPC-producing *K. pneumoniae* may be the result of failure of adequate intrahospital disinfection of medical surfaces and equipment. Wet environments (drains, faucets, sinks) are where these bacteria can survive and spread. Medical devices can be contaminated and, if not used properly, become vectors for spreading infections with these germs in hospitals. Initially, the bacteria can be transferred to the device via contaminated medical equipment, contaminated water, or other external environmental factors [6,7,8]. Furthermore, medical equipment and devices (e.g., endoscopes) can be contaminated and colonized with KPC-producing *Klebsiella pneumoniae* and, thus, become vectors of transmission of the infection from one patient to another [6]. Gastrointestinal endoscopes, despite the use of advanced disinfection techniques, can still harbor persistent contamination that increases the risk of bacterial transmission. Several factors contribute to this, including exposure to endogenous flora during procedures, the ability of bacteria such as *Klebsiella pneumoniae* to form biofilm on the endoscope surfaces, the design and surface characteristics of endoscopes that make thorough cleaning a challenge, and disinfection techniques that may not eliminate bacteria. The most identified bacteria associated with contamination, transmission, and infections associated with gastrointestinal endoscopes are *Klebsiella pneumoniae*, *Escherichia coli*, and *Pseudomonas aeruginosa* [6,9,10,11,12]. 

The increase in carbapenemase-producing *Klebsiella* species has led to high hospital mortality and limited treatment options. Hospitals worldwide implement strict measures to limit infection and prevent the spread of the bacterium but, currently, the effectiveness of these measures is still unknown [13,14,15,16]. Recently, more guidelines and recommendations have focused on controlling and preventing carbapenemase-producing *Enterobacteriaceae* infections. However, there is no agreement regarding the success of individual infection control measures regarding the spread of KPC-producing *Klebsiella pneumoniae* [13,17,18,19]. Several key issues must be addressed when treating KPC-producing *K. pneumoniae* in critically ill patients: prevention of colonization of the patient, prevention of infection in the colonized patient, prevention of colonization of the contacts of these patients, and reduction of mortality in infected patients [20].

Prolonged stay in the intensive care unit (ICU), dependence on invasive medical equipment, inappropriate antibiotic therapy, and chronic illness (diabetes, chronic obstructive pulmonary disease, cancer) are the most critical risk factors for the emergence of infections with KPC-producing bacteria. KPC-producing *Klebsiella pneumoniae* infections have also been associated with travel, immigration, and recent healthcare in areas where such infections are constantly present [3,21,22,23,24]. Intensive care units are mainly affected due to the multitude of maneuvers and invasive devices, but also due to the severity of the diagnoses of hospitalized patients and the large number of days of hospitalization [4,5,25]. Patients with bacteremia or respiratory infections due to carbapenemase-producing *Klebsiella pneumoniae* present a high death rate (30–70%) [4,5,25,26]. In our intensive care unit, we also face this major problem. KPC-producing *K. pneumoniae* is one of the most frequently isolated bacteria from samples collected from critical patients with extended stays; most of the samples were from tracheal aspirates (intubated and mechanically ventilated patients). We have found MDR (multidrug-resistant), XDR (extensively drug-resistant), and even PDR (pandrug-resistant) *Klebsiella pneumoniae* in our ICU.

**Figure 1:**
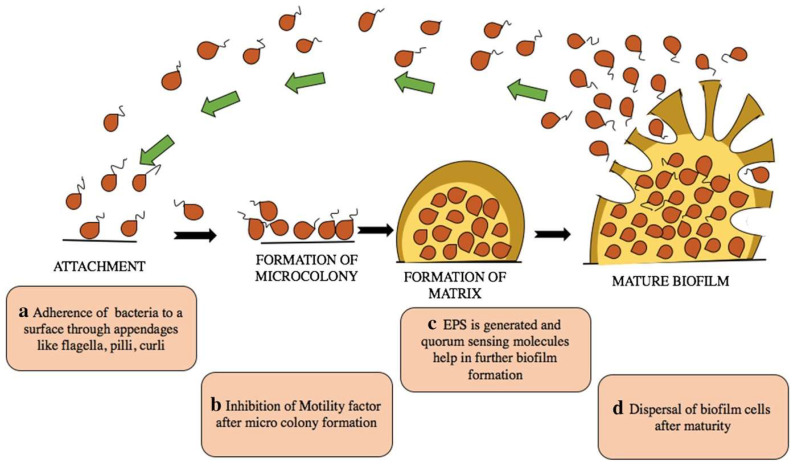
Stages of biofilm formation: adherence of bacteria to the surface (**a**); inhibition of motility factor (**b**); generation of EPS and quorum sensing leading to mature biofilm formation (**c**); dispersal of mature cells (**d**) [28].

Bacteria are single-celled organisms and attach to inert or living surfaces to form communities of microorganisms and biofilms. Bacterial biofilm is an aggregate of bacteria (belonging to one or more species of microorganisms) surrounded by a matrix they produce, adherent to each other and to surfaces and/or tissues. Microbes practically live on surfaces, including medical devices, leading to colonization and mature biofilm formation by secreting extracellular polymeric substances (EPS) that provide protection and resistance to aggressive factors, such as antibacterial agents (impossible for antibiotics to penetrate the biofilm), the host immune responses, and extreme environmental factors (UV radiation, extreme temperature, extreme pH, high pressure, high salinity, etc.) [27,28,29,30,31,32,33,34]. The biofilm formation steps are as follows: initially reversible attachment (adherence of bacteria to a surface), irreversible attachment (inhibition of motility factor and production of EPS), maturation, and dispersion (bacteria revert to their original form). Thus, the biofilm expands and establishes itself in new places, resulting in disease progression and the spread of infection (Figure 1) [28].

*Klebsiella pneumoniae* possesses the capacity to form biofilms. Gram-negative bacteria (including *Klebsiella pneumoniae*) produce acyl homoserine lactose inducer (AHL), which spreads out from the cell and enters another bacterial cell, attaches to, and activates the activator protein, binds to the DNA, and releases AHL synthetase, which catalyzes the creation of new AHL and process repeats, producing quorum sensing (cell–cell communication system) among the colony of microorganisms facilitating biofilm formation [28,35,36]. The phenotypic and genotypic characteristics of biofilm microorganisms differ from those of planktonic organisms, which confer strong resistance [27,28,29,30,31,32,37,38].

Multiple species of microorganism form biofilms. These biofilms are responsible for producing 80% of acute and chronic infections. While *Staphylococcus epidermidis* and *Staphylococcus aureus* are frequently associated with biofilm formation on medical devices, among *Staphylococcal* species, multidrug-resistant Gram-negative bacteria, especially *Pseudomonas aeruginosa*, *Klebsiella pneumoniae*, *Acinetobacter baumanii*, and *Escherichia coli*, are the most commonly involved in biofilm-based infections. *K. pneumoniae* is frequently associated with biofilms formed on central venous catheters (CVCs) and urinary catheters. *Staphylococcus aureus* biofilms are associated with post-implant orthopedic infections, chronic osteomyelitis, and endocarditis. *Pseudomonas aeruginosa* biofilms are usually responsible for catheter-associated urinary tract infections and contact-lens-related keratitis. *S. aureus*, *S. epidermidis*, *K. pneumonia*, *P. aeruginosa*, *Acinetobacter* spp., *E. coli*, and *Enterococcus* form biofilms on cardiovascular implants (prosthetic valves, pacemakers, and coronary artery bypass grafts). Methicillin-resistant *Staphylococcus aureus* (MRSA), *Klebsiella pneumoniae*, *Pseudomonas aeruginosa*, and *Acinetobacter baumanii* have been reported to colonize and form biofilms in endotracheal tubes and cause ventilator-associated pneumonia (VAP) [28,29,30].

Source control is the basis of treating infectious diseases and includes all the actions a multidisciplinary team takes in prevention and care [3]. Photocatalytic coatings are considered one of the best solutions for surface and medical device decontamination and self-disinfection, reducing the risk of infection transmission [39,40,41]. Titanium dioxide or Titania (TiO_2_) is one of the best photocatalytic materials for antimicrobial coatings. It has self-sterilizing effects and is considered a non-toxic material as a result of its inert nature compared to other metal oxides. In the presence of moisture and upon UV illumination, non-toxic metal oxides used for photocatalytic coating generate reactive oxygen species (ROS) (hydroxyl radicals, hydroperoxyl radicals, hydrogen peroxide, singlet oxygen, and superoxide radicals), kill microbes, and prevent their reactivation [39]. TiO_2_-coated surfaces could minimize bacterial adhesive interaction by changing the surface free energy and reducing bacterial adhesion by increasing the surface energy of the electron donor of the coating. Airborne ROS mobility could also destroy airborne microbes [39,42,43,44,45,46,47,48]. Most photocatalysts (including TiO_2_) are UV light absorbers. There is concern about using the entire solar spectrum, from UV to infrared wavelengths [49]. In several recent studies, TiO_2_ was combined with metals, non-metals, or other chemicals to enhance visible light absorption, its electron migration rate, and photocatalytic performance. Nanoparticles (NPs) of antimicrobial metals (titanium, gold, silver, zinc, copper) have antimicrobial and antibiofilm properties that are much better than their micro-sized counterparts [31,50,51,52,53,54,55]. Thus, TiO_2_ nanocomposite coatings find their applicability in hospital practice due to their essential antimicrobial role not only for surfaces but also for orthopedic and dental implants.

**Figure 2:**
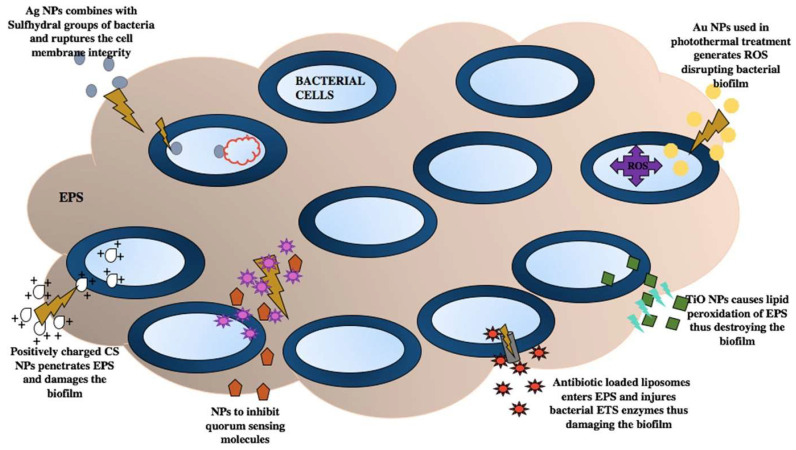
Mechanisms of NPs to combat biofilm: Ag-NPs damage bacterial DNA; Au-NPs produce ROS; TiO_2_ NPs cause lipid peroxidation of EPS; chitosan (CS) NPs destroy biofilm due to their positive charge; some NPs inhibit quorum sensing [28].

There is a severe threat posed by the ability of biofilms to grow and resist antibiotics. Various nanotechnology-based strategies have been studied and developed to prevent and combat serious health problems caused by biofilm infections. Factors such as mechanical stress, enzymatic digestion, oxygen availability, temperature, pH, and limited nutrition bring about the dispersal of bacteria from the biofilm, with bacteria becoming susceptible to antibiotics [56,57]. Nanoparticles (NPs) can play a vital antibiofilm role by EPS matrix disruption, dispersal of bacteria, and increasing susceptibility to antibiotics [57,58]. The NPs adopt various mechanisms to destroy biofilm. TiO_2_ NPs produce ROS in the bacterial cells, lipid peroxidation of EPS, cell membrane disruption, and the formation of extracellular polysaccharides (Figure 2) [28,48].

Only a few studies reported the use of NP-coated surfaces as antibiofilm agents. At the nanoscale, materials display distinct biological and physicochemical properties that their bulk counterparts do not. These unique properties are size-dependent, their dimensions being of the same order as biomolecules, and these materials can easily penetrate microbial cell walls and even biofilm EPS layers, causing irreversible DNA and cell membrane damage and, eventually, cell death. The large surface/volume ratios and long plasma half-lives improve their physicochemical reactivities and antibacterial and antibiofilm bioactivities. Other properties of nanoparticles identified as responsible for antibiofilm roles are given by shape, surface charge, and composition. The adherence of bacteria is inhibited by using surfaces with nano-roughness [28,29,59].

TiO_2_ nanoparticles demonstrated an excellent antibiofilm activity against bacteria (including *Klebsiella pneumoniae*) and fungi (*Candida albicans*) [26,28]. TiO_2_ NPs have been considered non-toxic materials compared with other metal oxides due to their inert nature. Silver (Ag) nanoparticles showed marked antibiofilm activity against *Pseudomonas aeruginosa*, *Escherichia coli*, *Klebsiella pneumoniae*, and *Staphylococcus aureus* (including methicillin-resistant *Staphylococcus aureus*). Prolonged exposure to Ag NPs may result in diminished efficacy, and excessive dosages may have toxic effects on skin cells. Zinc oxide (ZnO) nanoparticles exhibit good antibiofilm activity against *Staphylococcus aureus*, *Staphylococcus epidermidis*, *Escherichia coli*, and *Bacillus subtilis.* Copper oxide (CuO) NPs demonstrated antibacterial properties but to a lesser extent than Ag NPs or Zn NPs. Gold NPs showed little or no antimicrobial properties alone [28,29].

The International Agency for Research on Cancer (IARC) has recently classified titanium dioxide nanoparticles (TiO_2_ NPs) as possibly carcinogenic. Upon entering the body via inhalation, injection, dermal penetration, or gastrointestinal absorption, these particles could accumulate in various organs and induce harmful effects on cells and genes. The cytotoxic and genotoxic effects of TiO_2_ NPs are of particular concern, necessitating further research to ascertain the benefit–risk ratio associated with their use. As such, additional studies are required to assess the safety of TiO_2_ NPs and determine the optimal conditions for their use in various applications [60].

Conventionally, TiO_2_ nanoparticles are obtained by physical or chemical methods, using harmful reagents or energy-consuming manufacturing processes. Currently, there is an interest in the ecological synthesis of TiO_2_ NPs via biological methods using bacteria (*Acinetobacter baumannii* S1, *Acinetobacter seohaensis* N3, *Aeromonas hydrophila*, *Bacillus cereus* A1, *Bacillus mycoides*, *Rummeliibacillus pycnus* M1, and *Streptomyces* sp.), fungi (*Aspergillus flavus*, *Fomes fomentarius*, *Fomitopsis pinicola*, and *Trichoderma citrinoviride)*, or plant-based extracts (*Azadirachta indica*, *Ledebouria revoluta*, *Luffa acutangula*, *Mentha arvensis*, *Ocimum americanum*, *Piper betel*, *Prunus yedoensis*, and *Trigonella foenum-graecum*). Nanoparticles obtained through green, ecological technology have remarkable properties and dimensions and improved stability. Nanoparticles synthesized by biological methods mediated by bacteria are used in medicine as antimicrobial and anticancer agents due to their biocompatibility. Biogenic TiO_2_ nanoparticles have less applicability but are actively investigated [61,62,63].

The main aim was the evaluation of the implications of nanotechnology in eradicating biofilms with KPC-producing *Klebsiella pneumoniae*, one of the bacteria most frequently associated with nosocomial infections in intensive care units, including in our department. We also described the frequency of the presence of bacterial biofilms (including multidrug-resistant *K. pneumoniae*) on medical surfaces, devices, and equipment; the nosocomial dissemination of KPC-producing *K. pneumoniae*; and the importance of eradicating these biofilms, the main goal of the health system being to reduce patient morbidity and mortality and the costs associated with medical care. The secondary aim was to highlight studies presenting the potential applicability of TiO_2_ nanocomposite materials in hospital practice.

## 2. Materials and Methods

This review was conducted using the Preferred Reporting Items for Systematic Reviews and Meta-Analysis Guidelines 2020 (PRISMA). We used PubMed, Web of Science, Up to Date, and Cochrane Library as search engines. Between 2013 and 2024, over 300 specialized studies were published concerning the implications of antibacterial and antibiofilm nanocomposite coatings in relation to the role of TiO_2_ nanocomposite coatings in inactivation of carbapenemase-producing *Klebsiella pneumoniae*. We took into consideration meta-analysis, systematic reviews, and original studies. The keyword combinations used for searching the databases were: carbapenemase-producing *Klebsiella pneumoniae*, KPC-producing *Klebsiella pneumoniae*, TiO_2_ nanocomposite coatings, TiO_2_ nanoparticles, and biofilms.

The inclusion criteria for studies were reviews, meta-analyses, original studies, and peer-reviewed journals regarding MDR *Klebsiella pneumoniae* biofilm and the antimicrobial and antibiofilm effects of TiO_2_ nanocomposite coatings. Exclusion criteria were as follows: studies that are not on the subject of the theme addressed and single case reports.

## 3. Results and Discussions

When a search was conducted using the keyword combination “carbapenem-resistant *Klebsiella pneumoniae* biofilm”, 188 articles were identified. Using “carbapenemase-producing *Klebsiella pneumoniae* biofilm”, 76 articles were identified. When a search was conducted using the keyword combination “antimicrobial TiO_2_ nanocomposite coatings”, 76 articles were identified. The database search identified 19 articles when we used the keyword combination “TiO_2_ nanocomposite in photocatalytic inactivation of bacteria” and 39 articles when we searched for “antibiofilm nanocomposite coatings”.

**Figure 3:**
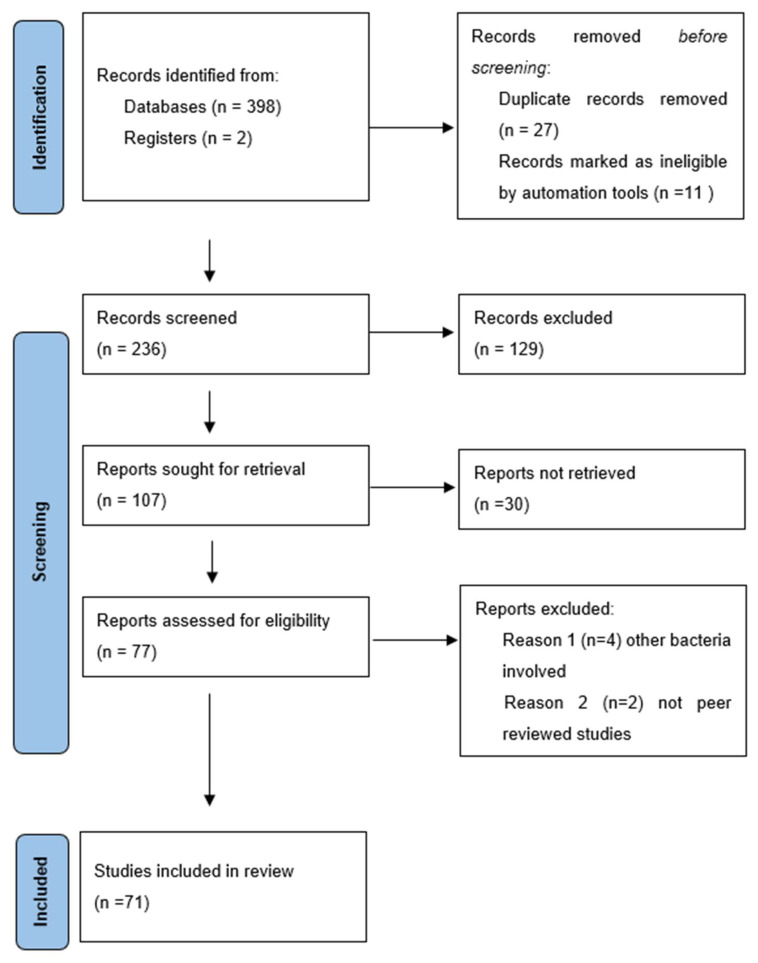
Research strategy of the present review.

The database search identified 398 records, including 27 duplicates. A total of 236 articles were selected for screening; 129 were excluded. Altogether, 77 articles were evaluated regarding eligibility, of which 71 were included in the final analysis; 61 were original reports, and 10 were reviews (Figure 3, Table 1).

Table 1 presents the characteristics of the 71 included studies. The studies included were divided into and discussed in two main categories: those focused on multidrug-resistant *Klebsiella pneumoniae* biofilm, and those investigating the antimicrobial and antibiofilm effects of TiO_2_ nanocomposite coatings. 

### 3.1. Studies on MDR Klebsiella Pneumoniae Biofilm

Studies describe a vital virulence trait used by *Klebsiella pneumoniae*: the ability to form biofilms on biotic and abiotic surfaces [64,65,66,67,68,69,70,71,72]. There are two types of biofilms: hydrated biofilms (in drains and catheters) and dry surface biofilms (DSB) (on surfaces and some medical devices). The biofilms lead to increased resistance to external stressors, antibiotics, and antimicrobial factors, and constitute an essential reservoir of pathogens, including MDR bacteria [73,74,75,76,77,78,79]. In their review, Banerjee and colleagues (2019) describe three hypotheses about the failure of the antibiotic susceptibility on the biofilm bacteria: failure of penetration of biofilm by bactericidal substance, altered chemical environment of biofilm, and altered gene expression of biofilm [28].

Folliero and colleagues (2021) reported that 72.7% of *Klebsiella pneumoniae* strains isolated from medical devices were biofilm-producing. They isolated the primary pathogens contaminating medical devices and studied their capacity to form biofilms and the prevalence of MDR-biofilm-producing strains. *Klebsiella pneumoniae* strains were detected in central venous catheters (CVCs), nephrostomy tubes, abdominal drain tubes, and Foley catheters. Some devices were colonized by more than one microorganism. Following analysis of the antibiotic susceptibility profiles detected of all isolated strains, 59.2% were MDR strains [80]. 

*Klebsiella pneumoniae* easily forms biofilms on catheters, nephrostomy tubes, abdominal drain tubes, Foley’s catheters, intubation cannulas, endoscopes, and other medical devices, but also on the hands of health professionals, and on intensive care unit environment surfaces [30,31,80,81,82,83]. The materials from which invasive medical devices and medical equipment are made, but also surfaces in patient rooms (e.g., furniture, paintwork) can influence the persistence of bacteria and the formation of biofilms. In their prospective study, Thorarinsdottir et al. showed the differences between the material of endotracheal tubes and biofilm formation. Compared to the uncoated polyvinyl chloride (PVC) endotracheal tubes, the noble-metal-coated PVC endotracheal tubes were associated with a lower rate of biofilm formation [82].

There are several biofilm-related infections, such as urinary-catheter-associated urinary tract infections, central-venous-catheter-associated bloodstream infections, and respiratory infections due to biofilms in the intubation cannula. Ventilator-associated pneumonia (VAP) is one of the most common intensive-care-unit- (ICU-) acquired infections occurring in patients who have been on mechanical ventilation for at least 48 h. The most common causes of VAP are bacteria (with an important role in MDR pathogens). The microorganisms most frequently isolated from these patients are aerobic Gram-negative bacteria (*Klebsiella pneumonia*, *Pseudomonas aeruginosa*, *Acinetobacter* spp., etc.) and, in more than 60% of the cases, Gram-positive cocci (particularly methicillin-resistant *Staphylococcus aureus*) [31,84,85,86,87].

Despite advanced disinfection methods, *Klebsiella pneumoniae* can survive on the surfaces of endoscopes, easily forming biofilms, which leads to the transmission of the bacteria from one patient to another, thus increasing the risk of infection. In a study conducted by Bourigault et al. in 2018, the phenomenon of cross-transmission during an outbreak of carbapenemase-producing *Klebsiella pneumoniae* was investigated. The study revealed a pattern wherein five patients were identified as having undergone endoscopic retrograde cholangiopancreatography (ERCP) procedures with the same endoscope. KPC-producing *Klebsiella pneumoniae* were identified in these patients and the duodenoscope was the only epidemiological link [10]. In a review published in 2020, Snyder describes that studies have demonstrated persistent gastrointestinal endoscope contamination despite appropriate and advanced disinfection techniques. Several factors contribute to endoscope contamination, including exposure to endogenous flora during procedures, the ability of bacteria such as *Klebsiella pneumoniae* to form biofilm on the endoscope surfaces, and the design and surface characteristics of endoscopes. The most commonly identified bacteria associated with contamination, transmission, and infections associated with endoscope are *Klebsiella pneumoniae*, *Escherichia coli*, and *Pseudomonas aeruginosa* [9].

The survival of *Klebsiella pneumoniae* poses questions about its persistence in healthcare settings. Dry surface biofilms (DSB) persist in the hospital environments, differ from the traditional “wet” biofilms, and represent a challenge for cleaning and disinfection. Biofilms in a dry state have recently been found to colonize dry surfaces such as ceilings, curtains, keyboards, door handles, light switches, trolley handles, ventilator inlets, mattresses, and bed rails. DSBs are challenging to remove due to increased resistance to disinfectants and detergents, and they periodically release bacteria that are a source of infection into the environment [88,89,90,91,92]. Hu and colleagues (2015) showed that ICU environmental surfaces are contaminated by MDR bacteria found in dry surface biofilms despite terminal disinfection with chlorine solution. Multiple species of microorganisms formed biofilms in 93% of samples. Polymicrobial biofilms are less susceptible to disinfection than mono-bacterial biofilms [91,93]. Centeleghe and colleagues published (2023) the first study that confirmed that *Klebsiella pneumoniae* can survive for a long time on dry surfaces as a dry surface biofilm (DSB). Although the culturability of *K. pneumoniae* from DSB is low after four weeks, the viability remains high. Transfer of bacteria from DSB is reduced over extended periods. After removing *Klebsiella pneumoniae* from surfaces by mechanical wiping and reducing culturability over time, the bacteria remained viable for up to 4 weeks of incubation, indicating viable but non-culturable status [94]. The biofilms on healthcare facility surfaces, especially high-touch surfaces, constitute an essential reservoir of pathogens and multidrug-resistant organisms, as dry surface biofilms persist for a long period of time and are difficult to clean and disinfect (Costa et al., 2019) [90].

### 3.2. Studies on Antimicrobial and Antibiofilm Effects of TiO_2_ Nanocomposite Coatings

Nanomaterials are used for biomedical applications, constituents of coatings, cancer treatment, tissue engineering, drug/gene delivery vehicles, and medical implants. Many studies describe the antimicrobial effects of TiO_2_ nanoparticles. Several reports have been conducted on photocatalytic biofilm inhibition by metal oxide nanoparticles, including TiO_2_ [29,84,95,96]. The antibacterial mechanism is linked to the ability of TiO_2_ NPs to produce ROS in microbial cells, lipid peroxidation of EPS, and oxidation of internal enzymes, which impairs cellular respiration and leads to apoptosis [39,97,98,99,100,101,102,103,104,105,106,107,108,109,110].

In December 2023, Pourmehdiabadi and colleagues published their study about the effects of ZnO and TiO_2_ NPs on forming biofilm and persister cells in *Klebsiella pneumoniae*. They investigated the expression of genes in *Klebsiella pneumoniae*, which has a role in bacterial persistence, under nanoparticle exposure and compared it with the expression of untreated bacteria as a control. They showed that another antibiofilm mechanism of NPs can be the change in gene expression in biofilm production [26].

There is an interest in using nanoparticles for antibacterial and anticancer properties, while avoiding their cytotoxic and genotoxic effects in long-term or invasive use (e.g., implants and invasive medical devices) [60]. Thus, the applicability of biogenic nanoparticles in medicine is being studied. Pandya and Ghosh published (February 2024) their study and reported that biogenic TiO_2_ NPs inhibit bacteria such as *Klebsiella pneumoniae*, *Escherichia coli*, *Pseudomonas aeruginosa*, *Proteus vulgaris*, *Salmonella enterica*, *Yersinia enterocolitica*, *Clostridium perfringens*, *Clostridium tetani*, *Streptococcus pneumoniae*, *Streptococcus pyogenes*, *Staphylococcus aureus*, *Enterococcus faecalis,* and *Vibrio cholerae* [63]. Biogenic, environmentally friendly, non-toxic nanoparticles have remarkable properties due to their biocompatibility, stability, and size. Biogenic TiO_2_ nanoparticles have less applicability but have been actively investigated [60,61,62,63,109]. Thakur et al. (2019) published a paper describing the antibacterial efficacy of the TiO_2_ nanoparticles against *Klebsiella pneumoniae*, *Escherichia coli*, *Staphylococcus aureus*, *Bacillus Subtilis*, and *Salmonella enterica* using *Azadirachta indica* leaf extract (green synthesis). This investigation showed that TiO_2_ nanoparticles (TiO_2_ NPs) prevented bacterial development. The minimum inhibitory concentration (MIC) of titanium dioxide nanoparticles against *K. pneumoniae* was 16.66 μg/mL and minimum bactericidal concentration (MBC) was 83.33 μg/mL (the lowest MBC value compared with *Escherichia coli*, *Salmonella enterica*, *Bacillus subtilis,* and *Staphylococcus aureus*). Titanium dioxide NPs showed the highest zone of inhibition (ZOI) 20.67 ± 1.45 mm at 200 μg/mL concentration against *K. pneumoniae* [109].

TiO_2_ coatings with active inorganic metals (like Ag or Cu), organic polymers, or 2D materials could demonstrate the maximum antibacterial efficacy compared to TiO_2_ or bare metals [29,98,100,103,104,105]. In their study (2016), Tahir and colleagues demonstrated that the Ag/TiO_2_ nanocomposite has a much higher photo inhibition activity against Gram-negative bacteria than bare TiO_2_ and Ag nanoparticles [111]. In their study, Naik and colleagues (2013) reported that mesoporous TiO_2_ nanoparticles containing Ag ions have excellent antimicrobial activity against Gram-negative and Gram-positive pathogens at low silver concentrations without photoactivation, and ensure long-term antibiofilm activity [112]. Bonan and colleagues (2019) investigated in vitro antimicrobial and anticancer activities of mesoporous and superhydrophilic TiO_2_ nanofibers containing AgNPs. They demonstrated that Ag/TiO_2_ nanofibers have antibacterial activity against Gram-negative and Gram-positive tested bacteria and strong potential for local cancer therapy [113]. Rahman and colleagues (2021) describe in their research work the influence of multimodal and flexible hybrid membranes that contain bacterial cellulose (BC) and photoactive (TiO_2_) and antibacterial (Ag) components (BC-SiO_2_-TiO_2_/Ag). This nano platform contained TiO_2_ and Ag with antibacterial properties and photocatalytic and self-cleaning characteristics, and showed significant antibacterial efficacy. These flexible membranes can be easily disinfected under UV irradiation and/or natural sunlight and can be used in different areas (antibacterial surfaces, filters, and face masks) [114].

There are not many large reviews on the antibacterial and antibiofilm applications of TiO_2_ photocatalytic nanocomposite coatings.

**Table 1:** Details of the papers identified through the systematic search.

Study	Country	Type of Study	Keywords
Alipanahpour Dil E et al., 2019 [53]	Iran	Experimental study	TiO_2_ nanocomposite coating
Araújo BF et al., 2018 [64]	Brazil	Cross-sectional	Biofilms, KPC-Kp
Aslam M et al., 2021 [62]	Malaysia	Review	TiO_2_ nanoparticles
Bai J et al., 2023 [77]	China	Cross-sectional	Biofilms, KPC-Kp
Banerjee D et al., 2019 [28]	India	Review	Biofilms, TiO_2_ nanocomposite coatings
Barani M et al., 2022 [95]	Iran	Review	Biofilms, nanocomposite coating
Bevacqua E et al., 2023 [60]	Italy	Review	TiO_2_ nanoparticles
Bode-Aluko et al., 2021 [40]	South Africa	Experimental study	Biofilms, nanocomposite coating
Booq RY et al., 2022 [68]	Saudi Arabia	Cross-sectional	Biofilms, KPC-Kp
Bonan RF et al., 2019 [113]	Brazil	Experimental study	Biofilms, nanocomposite coating
Bourigault et al., 2018 [10]	France	Experimental study	*Klebsiella pneumoniae*
Brunke MS et al., 2022 [7]	Germany	Case control	Biofilms, KPC-Kp
Cai Y et al., 2013 [101]	Sweden	Experimental study	Biofilms, TiO_2_ nanocomposite coatings
Centeleghe I et al., 2023 [94]	UK	Experimental study	Biofilms, KPC-Kp
Costa DM et al., 2019 [90]	Brazil	Cohort study	Biofilms, KPC-Kp
Dan B et al., 2023 [8]	China	Cohort study	Biofilms, KPC-Kp
D’Apolito D et al., 2020 [69]	Italy	Cohort study	Biofilms, KPC-Kp
Dey D et al., 2016 [76]	India	Experimental study	Biofilms, KPC-Kp
Fasciana T et al., 2021 [16]	Italy	Cohort study	Biofilms, KPC-Kp
Fetyan NAH et al., 2024 [61]	Egypt	Experimental study	TiO_2_ nanoparticles
Folliero V et al., 2021 [80]	Italy	Cohort study	Biofilms, KPC-Kp
Hebeish AA et al., 2013 [98]	Egypt	Experimental study	TiO_2_ nanocomposite coatings
Horváth E et al., 2020 [42]	Switzerland	Experimental study	TiO_2_ nanocomposite coatings
Hu H et al., 2015 [91]	Australia	Cross-sectional	Biofilm
Jones RN, 2010 [84]	USA	Cohort study	Biofilms, KPC-Kp
Joya YF et al., 2012 [106]	UK	Experimental study	TiO_2_ nanocomposite coatings
Kerbauy G et al., 2016 [74]	Brazil	Experimental study	Biofilms, KPC-Kp
Kumar A et al., 2017 [56]	India	Review	Biofilms
Kumaravel V et al., 2021 [39]	Ireland	Review	TiO_2_ nanocomposite coatings
Kiran ASK et al., 2018 [97]	India	Experimental study	TiO_2_ nanocomposite coatings
Ledwoch K et al., 2018 [89]	UK	Multicenter study	Biofilms
Liu Y et al., 2017 [70]	China	Experimental study	Biofilms, KPC-Kp
Lin Y et al., 2021 [102]	China	Experimental study	TiO_2_ nanocomposite coatings
Mahmud ZH et al., 2022 [71]	Bangladesh	Cohort study	Biofilms, KPC-Kp
Melsen WG et al., 2011 [86]	Netherlands	Systematic review	Biofilms, KPC-Kp
Mohammadi M et al., 2023 [87]	Iran	Cohort study	Biofilms, KPC-Kp
Moongraksathum B et al., 2019 [99]	Taiwan	Experimental study	TiO_2_ nanocomposite coatings
Motay M et al., 2020 [55]	France	Experimental study	TiO_2_ nanocomposite coatings
Mousavi SM et al., 2023 [59]	Iran	Experimental study	Biofilms, TiO_2_ nanocomposite coatings
Naik K et al., 2013 [112]	India	Experimental study	TiO_2_ nanocomposite coatings
Nica IC et al., 2017 [107]	Romania	Experimental study	TiO_2_ nanocomposite coatings
Nica IC et al., 2017 [108]	Romania	Experimental study	Biofilms, TiO_2_ nanocomposite coatings
Noreen et al., 2019 [105]	Pakistan	Experimental study	TiO_2_ nanocomposite coatings
Nosrati et al., 2017 [103]	Iran	Experimental study	TiO_2_ nanocomposite coatings
Ochońska et al., 2021 [83]	Poland	Experimental study	Carbapenemase-producing *Klebsiella pneumoniae*
Ohko et al., 2009 [110]	Japan	Experimental study	Biofilms, TiO_2_ nanocomposite coatings
Palacios et al., 2022 [75]	Spain	Experimental study	Carbapenemase-producing *Klebsiella pneumoniae*, biofilms
Pandya et al., 2024 [63]	India	Review	TiO_2_ nanoparticles
Papalini et al., 2020 [73]	Italy	Experimental study	KPC-producing *Klebsiella pneumoniae*, biofilms
Pourmehdiabadi et al., 2023 [26]	Iran	Experimental study	KPC-producing *Klebsiella pneumoniae*, biofilms
Prasad et al., 2019 [100]	India	Experimental study	TiO_2_ nanocomposite coatings
Rafiq et al., 2016 [37]	India	Experimental study	Carbapenemase-producing *Klebsiella pneumoniae*
Rahman et al., 2021 [114]	Pakistan	Experimental study	TiO_2_ nanocomposite coatings
Rani et al., 2021 [104]	India	Experimental study	TiO_2_ nanocomposite coatings
Sabenca et al., 2023 [81]	Portugal	Experimental study	Carbapenemase-producing *Klebsiella pneumoniae*, biofilms
Shadkam et al., 2021 [38]	Iran	Experimental study	Carbapenemase-producing *Klebsiella pneumoniae*, biofilms
Snyder et al., 2020 [9]	USA	Review	Biofilms
Silva et al., 2021 [72]	Brazil	Experimental study	KPC-producing *Klebsiella pneumoniae*
Singha et al., 2023 [54]	Bangladesh	Experimental study	TiO_2_ nanocomposite coatings
Stallbaum et al., 2021 [65]	Brazil	Cross-sectional study	Biofilms
Tahir et al., 2016 [111]	China	Experimental study	TiO_2_ nanocomposite coatings
Taylor et al., 2011 [96]	USA	Review	Biofilms
Thakur et al., 2019 [109]	India	Experimental study	TiO_2_ nanocomposite coatings
Thorarinsdottir et al., 2020 [82]	Sweden	Observational study	Biofilms
Veltri et al., 2019 [47]	Italy	Descriptive study	TiO_2_ nanocomposite coatings
Vickery et al., 2012 [88]	Australian	Experimental study	Biofilms
Yazgan et al., 2018 [67]	Turkey	Experimental study	Biofilms
Zhang et al., 2019 [43]	UK	Experimental study	TiO_2_ nanocomposite coatings
Zheng et al., 2020 [50]	Singapore	Experimental study	Biofilms
Zhou C et al., 2023 [79]	China	Experimental study	Carbapenemase-producing *Klebsiella pneumoniae*
Zhou H et al., 2021 [48]	China	Experimental study	TiO_2_ nanocomposite coatings

## 4. Conclusions

The degree of virulence of *Klebsiella pneumoniae* carbapenemase- (KPC-) producing *Klebsiella pneumoniae* has led scientists to identify new antibacterial compounds. Understanding the resistance mechanisms of *Klebsiella pneumoniae* can guide the development of new technologies to inhibit microbial growth and proliferation. *K. pneumoniae* is frequently associated with biofilms formed on central venous catheters, urinary catheters, and endotracheal tubes, but also endoscopes and different dry surfaces in the hospital. Recent developments in nanotechnology have significantly boosted the treatment of biofilm infections and proved promising for applications in removing pathogens. TiO_2_ photocatalytic coatings are one of the best long-term options for antimicrobial efficacy due to their biocompatibility, stability, corrosion resistance, and low cost. TiO_2_ nanoparticles demonstrated marked antibiofilm activity. TiO_2_ nanocomposite coatings with active inorganic metals, organic polymers, or 2D materials demonstrated the maximum antimicrobial and antibiofilm efficacy compared to TiO_2_ or bare metals. There are few comprehensive reviews regarding the antibacterial and antibiofilm applications of TiO_2_ photocatalytic nanocomposite coatings. This review summarized research studies on the role of nanomaterials, in particular TiO_2_ nanocomposite coatings, and their medical applications for preventing the spread of nosocomial infections with KPC-producing *Klebsiella pneumoniae*. The International Agency for Research on Cancer (IARC) has recently classified titanium dioxide nanoparticles (TiO_2_ NPs) as possibly carcinogenic. Currently, there is an interest in the ecological, non-toxic synthesis of TiO_2_ nanoparticles via biological methods. Biogenic, environmentally friendly, non-toxic nanoparticles have remarkable properties due to their biocompatibility, stability, and size. Research remains open in these areas, and questions regarding the interactions between nanoparticles, biofilm, and hosts, and their impact on natural systems need to be resolved. Biogenic TiO_2_ nanoparticles have less applicability but are actively investigated. Further research is needed to prevent and remove biofilm, to determine the safety and long-term effects of using metal nanoparticles as antimicrobial agents, and to ensure successful clinical applications.

## Data Availability

Data are contained within the article.

## Abbreviations

AHL	Acyl homoserine lactose inducer
Ag	Silver
Au	Gold
BC	Bacterial cellulose
Cu	Copper
CuO	Copper oxide
CVC	Central venous catheter
CRKP	Carbapenem-resistant *Klebsiella pneumoniae*
CS	Chitosan
DSB	Dry surface biofilms
EPS	Extracellular polymeric substances
ICU KP	Intensive care unit *Klebsiella pneumoniae*
KPC	Carbapenemase-producing *Klebsiella pneumoniae*
MBC	Minimum bactericidal concentration
MIC	Minimum inhibitory concentration
MDR	Multidrug-resistant
MRSA	Methicillin-resistant staphylococcus aureus
NPs	Nanoparticles
PDR	Pandrug-resistant
PRISMA	Preferred Reporting Items for Systematic Reviews and Meta-Analysis
PVC	Polyvinyl chloride
ROS	Reactive oxygen species
SiO_2_	Silicon dioxide
TiO_2_	Titanium dioxide
VAP	Ventilator-associated pneumonia
XDR	Extensively drug-resistant
ZnO	Zinc oxide
ZOI	Zone of inhibition
